# Letter to the Editor of *Obesity Pillars* from the American Board of Obesity Medicine

**DOI:** 10.1016/j.obpill.2023.100064

**Published:** 2023-04-19

**Authors:** Dana Brittan, Kimberly A. Gudzune, Edmond P. Wickham, Judith Korner

**Affiliations:** American Board of Obesity Medicine, Denver, CO, USA; American Board of Obesity Medicine, Denver, CO, USA; Johns Hopkins University School of Medicine, Baltimore, MD, USA; Virginia Commonwealth University School of Medicine, Richmond, VA, USA; a American Board of Obesity Medicine, Denver, CO, USA; American Board of Obesity Medicine, Denver, CO, USA; Columbia University Vagelos College of Physicians and Surgeons, New York, NY, USA

**Keywords:** Certification, Specialty boards, Obesity

To the Editor:

As representatives of the American Board of Obesity Medicine (ABOM), we would like to respond to “*Obesity Medicine as a Subspecialty and United States Certification – A Review*” [1]. ABOM is an independent organization solely focusing on certification of US and Canadian physicians in Obesity Medicine that adheres to Institute for Credentialing Excellence best practices. As a result, ABOM is regarded highly by organizations such as the American Medical Association and American Board of Medical Specialties (ABMS).

Since inception, US physician certification organizations have aimed to safeguard the public by setting high standards in determining competency [2]. While ABOM has an obligation to its Diplomates and the Obesity Medicine field, we must also consider how ABOM best serves the public and individuals with obesity. Given physicians’ well-documented gaps in obesity training [3], ABOM certification signifies acquisition of the specialized knowledge and skills required for the practice of Obesity Medicine. ABOM has certified nearly 7000 Obesity Medicine physicians [4] who provide care in every US state [5]. This evidence demonstrates the growing reach of ABOM Diplomates, even in the absence of ABMS membership. Research documents that the majority of ABOM Diplomates offer evidenced-based obesity care [6]. Therefore, as the number of Diplomates increases, ABOM anticipates improved access to high-quality clinical services for patients with obesity.

As interest in Obesity Medicine continues to grow, ABOM must carefully consider the potential benefits and consequences of Obesity Medicine becoming an ABMS subspecialty – for current Diplomates, patients, and the field overall. ABOM has met with various leaders from ABMS specialty and subspecialty boards to understand these issues and the requirements for recognition. ABMS subspecialty recognition remains a long-term prospect, but Obesity Medicine still has benchmarks to meet. For example, ABMS has historically required that there be at least 60 fellowship programs – a substantial increase from our current 24. Timeframe for subspecialty application remains uncertain, as meeting ABMS requirements cannot be accomplished by ABOM alone.

We appreciate the authors outlining the landscape regarding ABMS recognition for Obesity Medicine, and we would like to provide additional context for consideration. As obesity impacts patients of all ages who receive care across specialties, Obesity Medicine benefits from a diverse pipeline of primary specialties to address healthcare needs. However, ABMS structure does not easily accommodate cross-disciplinary fields. Although Obesity Medicine could potentially be a subspecialty under several ABMS boards, the specific board(s) cannot be determined until Obesity Medicine further matures. Under ABMS, the status of current ABOM Diplomates may be uncertain; not all ABOM-certified physicians would be eligible to become an ABMS Obesity Medicine specialist. A decreased certified Obesity Medicine physician workforce might negatively impact patient care and physician training.

As Obesity Medicine matures, ABOM will regularly engage ABMS regarding subspecialty recognition. Obesity Medicine has advanced over the last decade and will continue to coalesce as a distinguished practice area over coming years. In the meantime, ABOM will maintain the highest standards in physician certification – enhancing the legitimacy and prestige of Obesity Medicine now to support future endeavors.

## Declaration of competing interest

The authors declare the following financial interests/personal relationships which may be considered as potential competing interests: DB and KAG are the ABOM Executive Director and Medical Director, respectively. JK is Chair and EPW is Vice-Chair of the ABOM Board of Directors. KAG, EPW and JK are ABOM Diplomates. DB reports no other relationships. KAG also reports the following relationships: Novo Nordisk Inc (consulting/advisory; grant funding) and Eli Lilly and Company (consulting/advisory). EPW also reports the following relationships: 10.13039/100015519WW International Inc (grant funding); 10.13039/100011302Children's Hospital Foundation (grant funding); and Foundation for Advanced Education in the Sciences (speaking/lecture fees). JK also reports the following relationships: Gila Therapeutics (consulting/advisory); GI Dynamics (consulting/advisory); Found Health (consulting/advisory; equity/stocks); Digma Medical (equity/stocks); and Servier Laboratories (paid expert testimony).
